# Tertiary Lymphoid Organs in Cancer Immunology: Mechanisms and the New Strategy for Immunotherapy

**DOI:** 10.3389/fimmu.2019.01398

**Published:** 2019-06-20

**Authors:** Liangbin Lin, Xiang Hu, Huiyuan Zhang, Hongbo Hu

**Affiliations:** The State Key Laboratory of Biotherapy, Department of Rheumatology and Immunology, Collaboration and Innovation Center for Biotherapy, West China Hospital, Sichuan University, Chengdu, China

**Keywords:** tumor microenvironment, cancer immunology, tertiary lymphoid organ, non-canonical NF-κB, immunotherapy

## Abstract

The immune system plays pivotal roles in the occurrence and progression of cancers. As blockade of immune-checkpoint has been proven effective at improving anti-tumor immune response in multiple tumor types, the tumor immunotherapy still faces many challenges. Emerging evidence indicates lymphoid organ-like structures, also called tertiary lymphoid organs (TLOs) or ectopic lymphoid organs (ELOs), have been identified in cancers, as the result of lymphoid neoorganogenesis. The prognostic value of TLOs in cancer patients has been evaluated with debates, however, such well-organized lymphoid structures in the site of cancer indicate TLOs are the important modulators of cancer immunological microenvironment. TLOs have attracted remarkable efforts to investigate their neoorganogenesis and function in immune responses, aiming to develop new strategies for cancer immunotherapy. In this review, we summarize the current understandings about the molecular and cellular mechanisms governing the formation and function of TLOs in immune responses against cancer.

## Introduction

In the revised hallmarks of cancer that suggests a conceptual rationale, the importance of tumor microenvironment has been highly appreciated (1). The cancer cells do not manifest the disease alone; the collaborative interaction of neoplastic cancer cells and immune cells is crucial for tumorigenesis, local invasion, and metastases (2, 3). The immune cells that reside therein and those that migrate to the tumor in response to various signals are the key contributors of the tumor microenvironment (2). The remarkable achievements have been made to understand the function of immune cells in surveillance and clearance of cancer providing important insight into how these processes could be collaborative or misdirected in the context of cancer (4).

Most of the solid tumors have infiltrating immune cells; the presence of antigen-presenting dendritic cells (DCs) and lymphoid cells *in situ* indicates that such solid tumors could be recognized as the foreign and elicit an immune response (5). The presence of high numbers of tumor-infiltrating lymphocytes (TILs) has been considered as the prognostic. The basic observation or presumption of current immunotherapy strategies is that the immune system in tumor patients is impaired without efficient immune surveillance, so in general, many strategies have been developed to harness anti-tumor immune response, practically, blockade of immuno-checkpoints has been approved by FDA to treat several cancers (6, 7). Meanwhile, the immune cells provide an inflammatory milieu for tumorigenesis, progression, and metastasis (8, 9). The protumoral function of immune cells relies on the inflammatory milieu mediated by inflammatory cells for recruitment and induction of alternatively activated macrophage, myeloid-derived suppressor cells (MDSCs), and regulatory T cell (Treg) (10–12). Emerging evidence has indicated that even the same type or subpopulation of TILs sometimes has different or opposite effects on patient outcome, which becomes the greatest obstruction to design a tumor-immunotherapy approach (13, 14). However, the mechanisms driving this phenomenon are not fully understood to date.

**Figure 1:**
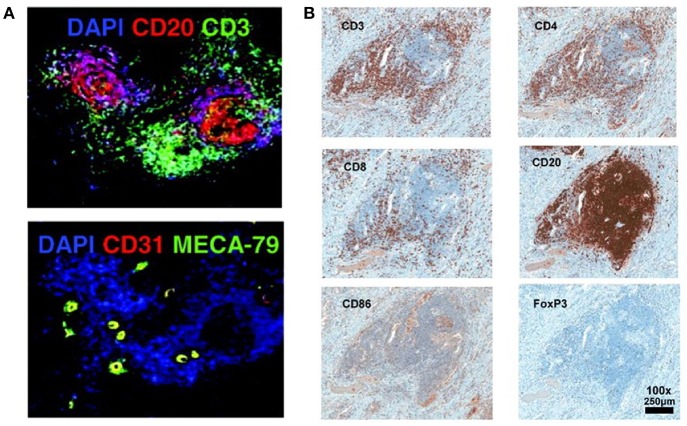
Histological structure and cell components of TLO detected by immunofluorescence or immunohistochemistry staining (20, 21). **(A)** T cells zone (CD3^+^), B cells zone (CD20^+^), and HEVs cells (CD31^+^ MECA-79^+^) are detected in the TLO of breast cancer tissue (20). **(B)** In melanoma associated TLO, CD20^+^ B cells form a follicle, with CD4^+^ and CD8^+^ T cells in the parafollicular cortex or marginal zones. CD86^+^ antigen presenting cells scatter the whole TLO structure. Only a few Foxp3^+^ Tregs are detected (21). The data/figures are cited from the indicated literatures with permission, all the right are reserved to the original publishers.

The lymph organs, referring the secondary lymphoid organs (SLOs), such as spleen, lymph nodes, Payer Patches, and mucosal-associated lymphoid tissue (MALT), etc., provide the three-dimensional structure for the optimized cell-cell interaction of different types of immune cells, to generate an effective immune response (15). Immune response could be triggered, independently of SLO, in tertiary lymphoid organs (TLOs) that develop under the chronic inflammatory condition, such as autoimmune disease, chronic infection, chronic graft rejection, and tumors (16–18). Similar to SLOs, the TLOs are histologically identified structures, characterized by presence of stromal cells, B-cell follicles, T-cell zones, and specialized vessels known as high endothelial venules (HEVs), although without encapsulating and afferent lymphatics (19). The typical histological structure and cell components of TLO detected by immunofluorescence or immunohistochemistry staining were showed in Figure 1 (20, 21). The spatial segregation of lymphocytes and stromal cells confers TLOs the potential capability to maintain a local immune response, which is suggested by the relevance of ectopic follicle formation to the diseases. In autoimmune non-obese diabetic mice, it would promote local production of autoantibodies (22). And in human autoimmune diseases, such as Hashimoto thyroiditis and Graves' disease, lymphoid follicles (LFs) were generated in thyroid gland which was assumed the main autoimmune response site (23). This intrathyroidal LFs are functional and might contribute to the expansion and perpetuation of autoimmune response (24). On the other hand, the development of TLOs may promote the eradication of pathogens and infectious agents, suggested by multiple animal studies with infection models (25, 26).

The function of TILs in the solid tumor is well-documented and it is generally accepted that the typical T-cell mediated anti-tumor immune response is initiated by DCs in the inflamed tumor microenvironment which ingest, process, and present the tumor-derived antigens to T-cell in SLOs, to activate the antigen specific anti-tumor T-cells (5, 27). T-cells, in turn, migrate back to the tumor tissue to eliminate the target antigen-expressing tumor cells. The presence of TLOs is correlated with better prognosis in many cancers including breast, lung, and colorectal cancer, however, the profound functions of TLOs in the immune response in tumor microenvironment are largely unknown (19, 28, 29). In this review, we will summarize the current knowledge of TLO in tumor microenvironment, and discuss the cellular and molecular mechanisms involved in the formation and function of TLO, and its potential to be a prognostic immune signature and novel immunotherapeutic target.

## The Function of TLO in Cancer

TLOs have been discovered for decades, mostly documented in autoimmune diseases, chronic infection, and rejection of organ transplantation. The immune response in these pathological settings is activated by persistent antigens released from the damaged tissues (30, 31). Since chronic inflammation is highly correlated with origin of tumors, which also provides the suitable microenvironment for neogenesis of TLOs, the prognostic value of TLO has been estimated in many types of tumors. Lymphocyte infiltration in tumor microenvironment, and the spatial aggregation in lymphoid structures are crucial in dictating patient outcomes. A great deal of studies has investigated the numbers, locations, frequencies, and cellular components of TLOs developed in tumor microenvironment, which indicates that the occurrence of TLOs is associated with better prognosis together with more infiltrating lymphocytes. Comparing to traditional haematoxylin and eosin (H&E)-staining which is used to identify lymphoid follicles, the immunohistochemistry-based methodologies to detect specific lymphocytes surface markers, combined with computer-based quantitative image assay, has provided a detailed assessment of the prognostic role of TLO subsets (32, 33). The prognostic relevance of TLOs in colorectal cancer, non-small-cell lung cancer, breast cancer, and melanoma cancer was summarized in Table 1.

**Table 1:** Prognostic relevance of tertiary lymphoid tissue in primary tumors.

**Type of cancer**	**TLT cellular composition**	**Prognostic marker**	**Methodology**	**Prognostic value**	**Number of patients**	**Tumor stage**	**References**
Colorectal	n.a.	H&E	H&E	Positive	843	I–IV	(34)
	T cells, B cells, FDCs	Gene signature	MA-GES	Positive	21	0–IV	(31)
	T cells, B cells, HEV, FDCs, DC-LAMP+DCs	CD3+ T cells	IHC	Positive	351	II–III	(35)
	T cells, B cells, CD68+macrophages, CD83+DCs	CD3+ CD20+ CD68+ CD83+	IHC	Positive	884	I–IV	(27)
	T cells, CD83+ DCs	CD3+ T cells, CD83+ DCs	IHC	Positive	40	I–IV	(36)
	CD8+ T cell, DC-LAMP+DCs	DC-Lamp+ mature DC	IHC	Positive	25	ND	(37)
Lung	T cells, B cells, DC-LAMP+DCs, FDCs	DC-LAMP+ mature DC	IHC	Positive	74	I–II	(38)
	T cells, B cells, DC-LAMP+ DCs, HEV	DC-LAMP+ mature DC,	IHC	Positive	362	I–IV	(39)
	T cells, B cells, DC-LAMP+ DCs, HEV, GCs	DC-LAMP+, CD20+ B cells	IHC	Positive	196	I–III	(40)
Breast	Tregs, T cells, DC-Lamp+ DCs	FoxP3+	IHC	Negative	191	I–III	(41)
	T cells, B cells	CD3+ T cells, CD20+ B cells	H&E, IHC	Positive	248	ND	(42)
	T cells, B cells, DC-LAMP+DCs, HEV	DC-LAMP+ DCs	IHC	Positive	146	I–III	(43)
	T cells, B cells	PNAD+ HEV	IHC	Positive	146	I–III	(20)
Melanoma	DC-LAMP+ DCs	DC-LAMP+	IHC	Positive	82	IA–IIIA	(44)
	DC-LAMP+ DCs	Gene signature	MA-GES	Positive	21	IV	(21)

*IHC, immunohistochemistry; Tfh, T helper follicular cells; GC, germinal center; FDCs, follicular dendritic cells; H&E, hematoxilin and eosin staining; DCs, dendritic cells; HEV, high endothelial venules; GES, gene expression signature; MA, microarray*.

### TLO in Colorectal Cancer

TLOs in human colorectal cancer (CRC) have been detected in multiple locations, intra-tumoral, and peritumoral regions, as well as at the invasive front of tumor. Many types of immune cells typically observed in SLO, including T-cell, B-cell, CD21^+^ follicular dendritic cell (FDC), and mature DC, together with CD31^+^ HEV and LYVE-1^+^ lymphatic vessels are found in TLOs (45, 46). In the CRC patients, both CD3^+^ TLOs and TILs are prognostic biomarkers in both primary and metastatic CRC (47). T cell-enriched TLOs and TILs are correlated with immune components identified in low-risk CRC, so the immunological events involved in tumor rejection are enhanced in local tumor microenvironment with TLO occurrence (31, 48). TLO frequency correlates with immune cells infiltration which is coordinated in better prognosis of stage II CRC patients (34, 36, 37). In the colitis-associated cancer animal model, B-cell follicle formation is observed in the sites of chronic inflammation associated with intestinal neoplasia (49). Since chronic inflammation is involved in CRC tumorigenesis, it is difficult to tell whether TLOs contribute to persistence of tumor-associated inflammatory reaction or participate in anti-tumor response. Another animal study indicates that adoptive transferred GFP-positive splenocytes by intravenous injection results in homing of those lymphocytes into TLOs, suggesting TLOs might be the sites for immune cell migrating to mount an efficient anti-tumor immune response in tumor area (35).

### TLO in Non-small-cell Lung Cancer

In human non-small-cell lung cancer (NSCLC) specimens, the presence of TLO structures is also correlated with clinical outcomes (38). The cellular components of TLOs are similar to those in SLO, with mature DC/T-cell zones adjacent to B-cell follicles, indication of activated immune responses. The density of mature DCs (DC-LAMP+) is highly correlated with the density of infiltrating CD4^+^ and T-bet^+^ T-cell into tumor, which also is associated with a favorable long-time survival of patients (38). While T-cells, both naive and central memory CD4^+^ and CD8^+^ cells, are identified in TLOs, the overall T-cell infiltration and density are less important than the density of mature DCs in TLOs since the patients with high DC-LAMP^+^ mDC have a dramatically improved clinical outcome (39).The infiltration of DCs might be controlled by CXCL12 and CXCR4 interaction, suggested by gene array analysis (50). As for B-cell, the germinal center-like structure may not exist in some cases, however, TLOs are the local sites for priming and expansion of both B- and T-cells (51). The prognostic value of B-cell seems to be more dramatic which also provides a protective immune response against lung cancer. Histologically, B-cell follicles aggregating with CD21^+^ FDCs, form germinal-center like structures (38, 52). These B-cells mediate a tumor associated antigen specific antibodies production locally, suggesting the B-cells are also involved in the humoral immune response against lung cancer (40). Additionally, the antitumor immune response could be suppressed by Tregs within tumor-associated TLS (TA-TLS) in a mouse model of lung cancer (53).

### TLO in Breast Cancer

The presence of TLO is frequently correlated with better clinical outcome in HER-2 positive and triple-negative breast cancer (TNBC) (54). Both the density and spatial organization of TLO are the determining factors of their prognostic value. A recently report suggests that TLO is associated with higher tumor grade, lymphovascular invasion, and more TILs, as well as hormone receptors negativity, HER2 positivity, and *c-kit* expression (42). TLO is dramatically related to better disease-free survival in HER2^+^ breast cancer, which is independent of TIL status, indicating TLO, and TIL might be the independent favorable factors associated with disease-free survival in these cases (42). Two independent studies suggest that HEV in TLOs related with T- and B- lymphocyte infiltration and have favorable prognosis in breast cancer (20, 43). Foxp3^+^ Tregs infiltration in TLO indicates the high risk of disease relapse and death in primary breast cancers (41).

### TLO in Melanoma Cancer

The presence of TLO in human primary melanoma is associated with better prognosis. Immunohistochemical analysis of 82 patients with cutaneous malignant melanoma shows that presence of peritumoral DC-LAMP^+^ mature DCs combined with OX40^+^ activated T cells, suggesting a functional immune response, associate with significantly longer survival (44). In the case of metastatic melanoma, TLOs identified in patients have more profound structures, including T-zones surrounded by mature DCs, distinct B-zone, HEV, and germinal centers. Strong B-cell mediated antibody production specific against melanoma has been provoked indicated expression of (activation-induced cytidine deaminase (AID), the enzyme which is required for somatic hypermutation of Ig and affinity maturation (55). Chemokine expression in melanoma cell are critical for TLO induction, indeed, analysis of 12-chemokine gene expression signature (GES) on genomic arrays of 14,492 solid tumor samples revealed that the presence of TLO directly correlates with the expression level of these 12-chemokine GES score (21). However, formation of TLO surrounding tumor might be immune suppressive. Recruitment of CCR7^+^ lymphoid tissue inducer (LTi) cells by CCL21-expressing melanomas leads to TLO formation, which also recruit immune suppressive cells like CD4^+^ Treg cells and MDSCs to suppress anti-tumor immune response (56). Further study is required to illuminate the key factor(s) to induce anti-tumor, but not immune suppressive microenvironment of TLO.

## Molecular Mechanisms of TLO Development and Function

**Figure 2:**
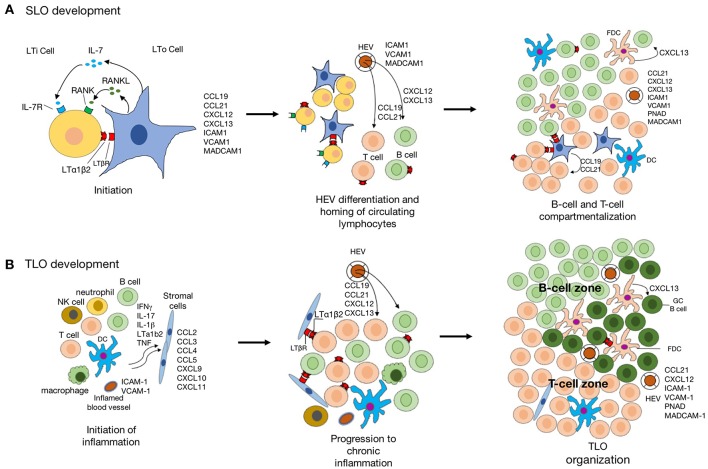
The schematic diagram indicating the development of SLO and TLO. **(A)** Schematic model of SLO lymphoid organogenesis. The interaction lymphoid tissue organizer (LTo) and lymphoid tissue inducer (LTi) is crucial for the initiation of SLO development. The signaling pathways activated by LTβR-lymphotoxin-α1β2 (LTα1β2), RANK-RANKL, and IL-7-IL-7R lead to the expression of a group of chemokines and adhesion molecules. The formation of high endothelial venules (HEVs) facilities the recruitment of naïve lymphocytes from circulation. Further, CCL19 and CCL21 produced by stromal cells regulate the homing of CCR7^+^ T cells and migration DCs to T-cell areas, whereas CXCL13 produced by follicular dendritic cells (FDC)s and DCs in germinal center attract CXCR5^+^ B cells into the follicles. **(B)** The inflammatory response is mediated by various innate immune cells (such as macrophages and DCs), leading to the recruitment of lymphocytes into the inflamed tissue. stromal cell in inflamed tissue are also activated to produce chemokine for lymphocyte recruitment which is suppressed when the inflammation is resolved. However, the chronic inflammation leads to activation of innate and adaptive immune cells in the inflamed tissue with expression of LTα1β2 by activated B- and T-cells, and lymphoid chemokines expressed by resident stromal cells, infiltrating macrophages, DCs, and other parenchymal cells. Recruitment of B cells, T cells, and DCs to TLO is facilitated by acquisition of a HEV-like phenotype by activated endothelial cells. CCL19 and CCL21 produced by stromal cells promote the formation of T-cell zone. Activated by LTα1β2, stromal cells acquire the phenotypic and functional features of FDCs and promote GC organization.

In the regard to the similarity of structure and function between SLO and TLO, our knowledge of TLO development derived from studies of SLO organogenesis. The initialization of SLO development is the presence of LTi and lymphoid tissue organizer (LTo), followed by the dynamic interaction between hematopoietic cell and stromal cell. As for TLO development, which is related to chronic inflammation often the time, the inflammatory cytokine-activated signal pathways are critical, besides the shared signal pathways involved in SLO development (Figure 2).

### Inflammatory Factors

**Figure 3:**
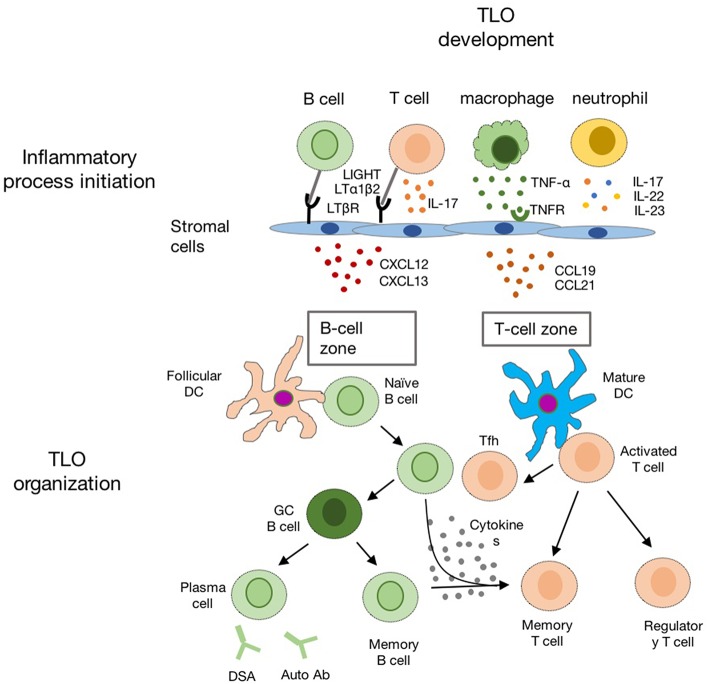
The schematic diagram indicating the development of TLO in the chronic inflammatory conditions. Chronic inflammation leads to activation of innate and adaptive immune cells (such as macrophages, neutrophil, T-, and B-cells) in the inflamed tissue. Multiple chemokines (such as CXCL12, CXCL13, CCL19, and CCL21) are released by activated local stromal cells to recruit T-, B-cells, and DCs for TLO formation with distinct B cell zone and T cell zone.

The chronic inflammatory microenvironment provides the initial signal for TLO formation, which is the major difference between TLO and SLO in development (57). The over-expression of TNFα in mice could overcome the deficiency of LTi cell to induce SLO formation (58). In regard to TLO, it is also notable that supporting microenvironments for TLO development are very devised, since TLO could be raised in different scenarios. Studies using different animal models suggest that ILC3/LTi are not always essential for TLO development, and chronic inflammation is sufficient to induce TLO formation (59–61) (Figure 3).

IL-17 is an inflammatory cytokine family with six members to activates the MAPK, NF-κB, PI3K, and C/EBP signal pathways functions through a heterotrimeric receptor of two IL-17RA and one IL-17RC subunit (62). The function of IL-17 in immune response is involved in host defense, inflammation and autoimmunity. IL-17 is mainly produced by Th17 cell, as well as by γδT-cell and ILC3 (LTi). Indeed, LTi and Th17 share some crucial developmental and phenotypic characters. RORγt and AHR are essential transcription factors to induce the development and maintain their function of both Th17 and LTi. Both of them also express CCR6^+^ and IL-7R for cell migration and survival (63). Given the similarity of LTi and Th17 cells, Th-17 directly contributes to lymphoneogenesis and development of TLOs (64). In the T-cell transfer induced experimental autoimmune encephalomyelitis (EAE) model, the Th17 cells infiltrated in the central nervous system induced the TLO formation in the subarachnoid space. As for the infection-associated TLO formation (iBALT), IL-17 produced by Th17 alone overruns the absence of LTi cell and mediates formation of iBALT (65). However, studies indicate iBALT formation might also have IL-17-independent mechanism, since iBALT formation is normal in RORγt KO, IL-17A, and IL-17F double deficient mice (66).

IL-22 is Th17-related cytokine which is not only produced by Th17, but also by LTi. IL-22-/-mice have significant defect in early TLO maturation, and abolished lymphoid chemokine expression. In the virus-induced TLO formation model, IL-22 signaling is necessary for CXCL12 and CXCL13 expression in epithelial and fibroblastic stromal cells, which leads to B-cell recruitment and TLO formation (67). Colonic lymphoid patches (CLPs) and isolated lymphoid follicles (ILFs) are TLOs in colon induced by chronic infection. In the *Citrobacter rodentium* infection model, IL-22 induced by LTα1β2 is required for the organization and maintenance of mature CLPs and ILFs in colon (68). Blockade of either the IL-22 or LTα1β2 pathway significantly reduced the number of ILFs, and IL-22 may function similarly in the formation of ectopic lymphoid follicles in other tissues during inflammation (68). It also possible that IL-22 and lymphotoxin synergistically contribute to chemokine production, thus coregulating the enlargement, organization, and maintenance of the inflammatory aggregates.

### Non-canonical NF-κB in the Development of TLOs

NF-κB is a family of transcriptional factors that plays critical roles in various biological processes. According to the different activating mechanisms, NF-κB could be divided into canonical and non-canonical NF-κB pathway, both of which are important for TLO development (69). The canonical NF-κB is often associated with inflammation, which is discussed above; meanwhile the non-canonical NF-κB is involved in the development and homeostasis. Compared to the transient and robust activation of canonical NF-κB pathway, the activation of non-canonical NF-κB pathway is slow but persistent, which is correlated with their biological functions (70).

The function non-canonical NF-κB pathway in the development and architectural organization of SLOs, including spleen, lymph nodes, and mucosal lymphoid tissues, is well-established using the transgenic or gene knockout mice models (71). Loss-of-function of NIK, and deficiency of downstream signaling components impairs the development of lymph nodes, PPs, and disturbed spleen architecture (72). LTα1β2, the well-defined ligand of LTβR, has essential function in lymphoid organ development, by activating non-canonical NF-κB pathway. This cytokine predominately produced by LTi, functions through LTβR expressed on LTo. LTα1β2-induced non-canonical NF-κB (p52/RelB) pathway is essential for chemokine (CXCL13, CCL21, and CCL19) and adhesion molecules (MAdCAM-1) expression by LTo, which mediates the recruitment of immune cells as well as LTi for the growth and development of lymph nodes (73–75).

The functions of lymphotoxin dependent pathway in TLO formation have been examined. Ectopic expression of LTα, under the control of rat insulin promoter II (RIP) causes the formation of TLO in pancreas and kidney, with accumulated T and B cell, DC, macrophages, HEV, and lymphoid chemokine expression (76). This lymphangiogenesis might be more inflammation-related, since overexpressed LTα forms LTα3 homotrimer and activates TNFR1 dependent signaling pathway. In the aged NOD (non-obase diabetic) and *ApoE*^−/−^ mice, LTβR mediated non-canonical NF-κB pathway induces CXCL13, CCL21, and MAdCAM-1 expression, to recruit and activate immune cells forming TLOs in metabolic diseases condition (77).

Several strategies have been developed to induce TLO formation for cancer immunotherapy by activating LTβR signaling pathways. Administration of agonistic antibody against LTβR promotes immune cell infiltration into tumor tissues and anti-tumor immunity (78). Overexpressed LIGHT (another ligand of LTβR) in tumor cells promotes TLO formation, with enhanced T-cell mediated anti-tumor immune response (79). Another study reported that mesenchymal stem cells with overexpressed LIGHT could migrant into tumor sites and cause tumor regression (80). The strategies above highlight the critical role of LTβR-activated non-canonical NF-κB pathway in TLO formation and functions in anti-tumor immunity. LTα1β2 also induce IL-22 production by LTi during *C. rodentium* infection, but LIGHT fails to do (81).

### IL-7 in TLO Development

IL-7 is responsible for development of multiple immune cells and immune homeostasis. The IL-7 receptor is heterodimer, consisting of IL-7 receptor alpha chain (IL-7Rα) and a common gamma chain (γC). The binding of IL-7 and its receptor complex activates JAK3-STAT5, PI3K-AKT, and mTOR pathway (82). The function of IL-7 in formation of SLO is well-established. Several types of lymph nodes were hardly detectable in IL-7 and IL-7Rα knockout mice, which is not simply because of deficiency of T- and B-cell. VCAM1^+^ICAM1^+^ mesenchymal cells and lymphatic endothelial cells are important source of IL-7 for LTi survival (50). Additionally, IL-7, or together with RANKL, induces LTi to secrete LTα1β2, which further interact with LTβR of stromal cell to promote the maturation of LTo and chemokine production (CXCL13, CCL19, and CCL21) for lymphocyte recruitment (83). Consistently, in the transgenic mice model with overexpressed IL-7, the CD4^−^CD3^+^IL-7Rα^+^ LTi is accumulated leading to the increased PPs numbers and formation of multiple TLOs. This phenotype is attenuated by deficiency of RORγt or LTα1β2, suggesting RORγt is the key factor for LTi linage dependent, meanwhile IL-7 signal is for LTi survival (83).

Emerging evidence suggested that IL-7 is also critical for TLO development. IL-7 transgenic mice develop spontaneous TLOs, and chronic inflammation such as chronic colitis, dermatitis (84). Studies from RA patients indicated that expression level of IL-7, IL-7R, and JAK3 were significantly increased in the samples with TLOs, compared to tissues without non-organized T- and B-cell infiltration (85, 86). IL-7 promotes monocytes, macrophage, and dendritic cell to produce more chemokine, adhesion molecule, and co-stimulatory molecules to enhance pro-inflammatory effector T-cell function, which correlates with clinical symptoms in RA patients (87). Collectively, IL-7 promotes inflammatory response mediated by T-cell and macrophage, and TLO formation.

## TLO as the New Therapeutic Target of Cancer Immunotherapy

As discussed above, TLO could serve as the powerhouse for anti-tumor immune response. Interestingly, TLO could be induced by traditional immunotherapy, in those cases, TLO are considered to orchestrate immune cell infiltration and activation to generate an immunogenic microenvironment to eliminate tumors (88). In the clinical trial with irradiated granulocyte-macrophage colony stimulating factor (GM-CSF)-secreting pancreatic tumor vaccine (GVAX), intratumoral TLO formation was observed in most of patients enrolled.

To design a clinical potent immune therapy for solid tumor, it is crucial to enhance the delivery of targeted lymphocytes to tumor sites and to ensure the sufficient expansion of activated lymphocytes in the immunosuppressive tumor microenvironment. A bioactive polymer implant near tumor tissue could facilitate the delivery, expanding, and dispersing of tumor-reactive T cells, which has been proved to cause the regression in a multifocal ovarian cancer model (89). This method might be valuable for CAR-T or TCR-T therapy for the solid tumor, since the major challenge of the adoptive transfer of CAR-T or TCR-T is that defective mobilization of T cells to the tumor sites.

Ganss's group established a *de novo* method to induce intratrumoral TLO and vessel normalization which could enhance immunotherapy in resistant tumors (88). A compound composed of mouse LIGHT protein and carboxy-terminal vascular targeting peptide (VTP) was designed to specific introduce LIGHT signal to tumor vessel, to induce intra-tumoral TLO formation. After LIGHT-VTP injection, TLOs were developed in majority of solid tumors, detected by immunohistochemistry and immunofluorescence. Among of group of pro-inflammatory cytokine and chemokine, IL-6, IL-1β, and CCL21 are critical for TLO induction. Adaptive transfer experiments indicate that macrophage and T-cell are required for the formation and maintain of TLO. *In vivo* studies show that LIGHT-VTP leads to anti-tumor immune response, with more immune cells infiltrating into tumor sites and induction of more effector and memory T-cells. Although LIGHT-VTP increases the efficacy of checkpoint-blockade therapy, the anti-tumor immune response is maximized when combined with tumor vaccine and checkpoint-blockade therapy, LIGHT-VTP therapy dramatically enhances anti-tumor immune response (88).

Similar strategy has been used in the study using a fusion protein containing LIGHT and anti-EGRF antibody which could specifically target EGFR^+^ tumor cell (90). By activating LTβR in stroma cell, this treatment up-regulates the expression of various cytokine and chemokine in tumor microenvironment, resulting in the increased T-cell infiltration and T cell-inflamed tumor microenvironment. Interestingly, the fusion protein synergizes with immune checkpoint blockade therapy (90). Taken together, TLO induction combined with immune checkpoint blockade could maximize anti-tumor effect with better outcome.

## Prospect

A great amount of experimental and clinical studies has established TLOs as the functional immune organs to recruit and activate T cells in the tumor site, mediating an effective anti-tumor immune response. These experiments established that induction of a lymphoid neogenesis favorable environment in tumor tissue could be effective in local T-cell response and tumor regression; thus, the key players of molecular pathways of TLO development are the promising targets to induce TLO as the alternative immunotherapeutic strategies. However, there are several questions need to be answered: what are the reasons that the TLOs develop only in certain portion of tumor patients, but not all? Where do TILs migrate from before and after TLO neogenesis? Could TLO be a boot camp for TILs? After all, the occurrence of TLO could the result of that immune system exerts an effective anti-tumor activity, in this scenario, TLO has been proposed as a very promising strategy to promote the delivery of an effective T cells into inaccessible areas of solid tumors.

## Author Contributions

LL and XH prepared the figures. HH and HZ contributed equally to the designing and writing of the review article.

### Conflict of Interest Statement

The authors declare that the research was conducted in the absence of any commercial or financial relationships that could be construed as a potential conflict of interest.
